# Evaluation of a novel body temperature sensor (CORE) in dogs under general anesthesia following premedication with dexmedetomidine or acepromazine: A pilot study

**DOI:** 10.1371/journal.pone.0360808

**Published:** 2026-10-15

**Authors:** Chloe R. Dupleix, Jacob P. Rastas, Qianqian Zhao, Robert Young, Rebecca A. Johnson

**Affiliations:** 1 Department of Surgical Sciences, University of Wisconsin, Madison, Wisconsin, United States of America; 2 Department of Biostatistics and Medical Informatics, University of Wisconsin, Madison, Wisconsin, United States of America; 3 WaldoPet, Waukesha, Wisconsin, United States of America; University of Messina, ITALY

## Abstract

Core body temperature monitoring in anesthetized dogs is a vital part of physiological evaluation. Depending on the procedure and patient positioning, consistent core temperature measurements can be difficult to obtain. The objective of this study was to evaluate the reliability and clinical validity of a wireless, non-invasive, superficial heat flux-based thermometric device (CORE, greenTEG AG, Rümlang, Switzerland). Three healthy beagle dogs were premedicated with either dexmedetomidine or acepromazine prior to induction with intravenous propofol and isoflurane maintenance. They were monitored every five minutes with pulse oximetry, non-invasive blood pressure, electrocardiography, and capnometry. Rectal thermometry and temperatures from the CORE sensor placed on clipped and unclipped skin over the jugular veins were measured every three minutes for up to 120 minutes following induction. Based on the correlation coefficient for repeated measures (*r*_*rm*_) and the Shrout-Fleiss intraclass correlation coefficient (ICC), in dogs administered acepromazine, there was good, statistically significant correlation and reliability between rectal and clipped CORE temperatures (*r*_*rm*_ = 0.81, P < 0.0001; ICC = 0.78) and rectal and unclipped CORE temperatures (*r*_*rm*_ = 0.79, P < 0.0001; ICC = 0.85). In dogs administered dexmedetomidine, although values were correlated (*r*_*rm*_ = 0.30, P < 0.0001), there was poor reliability (ICC = 0.28) between rectal and clipped CORE temperatures which improved if dogs were unclipped (*r*_*rm*_ = 0.48, P < 0.0001; ICC = 0.88). Clinically acceptable Bland-Altman agreements were found between rectal and unclipped CORE methods. These preliminary results suggest that the CORE sensor may provide an alternative method for assessing core body temperatures in unclipped, anesthetized dogs.

## Introduction

Hypothermia and impaired thermoregulation are common complications of general anesthesia in dogs, partly attributable to anesthetic drugs which may impair hypothalamic thermoregulatory centers as well as autonomic responses such as vasoconstriction [1,2]. In addition, acepromazine, an alpha-1 adrenergic receptor antagonist, and inhaled anesthetics such as isoflurane, decrease systemic vascular resistance [3–5] which may exacerbate heat loss, especially in clipped animals as fur is a natural insulator. Conversely, dexmedetomidine, an alpha-2 adrenergic receptor agonist, causes peripheral vasoconstriction that may limit heat loss and attenuate decreases in body temperature during anesthesia [6].

Currently, digital rectal thermometry in dogs is considered an acceptable estimate of core temperature [7]. However, mammalian core temperatures can be several degrees higher than surface temperatures, indicating that temperatures are not distributed uniformly. These differences may be enhanced during anesthesia due to redistribution of blood flow patterns and further heat transfer via evaporation, convection, radiation, and conduction [2]. In addition, rectal thermometry has limited usefulness for chronic temperature monitoring and may not be an easily accessible site during some anesthetized procedures. Thus, several thermometric methods such as infrared thermometry have been proposed as alternatives with variable results in conscious and anesthetized dogs [8–10]. Recently, a novel, non-invasive skin sensor (CORE apparatus; greenTEG AG, Rümlang, Switzerland) that uses machine learning algorithms based on measurements of heat flux transfer to continuously determine temperature has become commercially available [11]. Although its reliability and validity have had mixed results in humans, it has not been investigated in animals, and no studies exist to compare the reliability and clinical validity of the use of the CORE sensor in conscious or anesthetized dogs [12–14].

Our aim was to determine the reliability, correlation, and clinical validity of the CORE thermometric sensor compared with digital rectal thermometry in dogs administered either acepromazine or dexmedetomidine prior to undergoing inhalant anesthesia. The influence of skin condition (clipped versus unclipped) on sensor contact was also investigated. Our hypothesis was that following either acepromazine or dexmedetomidine administered prior to inhalant anesthesia in dogs, the reliability and clinical validity of the CORE sensor placed on clipped skin would be statistically reliable and clinically acceptable, but when placed on unclipped skin, these relationships would become non-significant since fur might interfere with sensor contact.

## Materials and methods

### Animals

All procedures and protocols were approved by the University of Wisconsin’s Institutional Animal Care and Use Committee (Protocol Number: V006724) and this study was performed in strict accordance with the Guide for the Care and Use of Laboratory Animals of the National Institutes of Health. Three healthy male beagle dogs aged approximately 8-months with a mean body weight of 8.5 ± 0.6 kg (mean ± standard deviation) were used; they were enrolled in a concurrent study investigating warming devices during general anesthesia in dogs [15]. At the conclusion of the study, no dogs were euthanized and they were adopted out in accordance with University of Wisconsin-Madison’s Policy UW-4102: Animal Adoption. All efforts to alleviate animal suffering were made during the procedures. Physical examinations, packed cell volume, total protein, and blood urea nitrogen (Azostix; Siemens Healthcare Diagnostics Inc., Tarrytown, NY) were performed before anesthesia to deem each patient healthy.

Prior to the study, all dogs were acclimated to their housing environment for seven days. Dogs were randomly assigned to protocols using SAS Software (Cary, NC) in a crossover design with two anesthetic periods using different pre-medicants (acepromazine, dexmedetomidine). Prior to anesthesia, a 5 cm section of fur over the left jugular vein was clipped. The three thermometric groups were described as clipped, unclipped, and rectal.

### Temperature monitoring techniques

Two CORE sensors were threaded over thin fabric with hook-and-loop closure that was used as a collar around the dog’s neck. One sensor was positioned over the left-sided clipped jugular vein and the second was positioned over the right-sided unclipped jugular area. A digital rectal thermometer (YSI 4600, YSI Incorporated, Yellow Springs, OH; accuracy = 0.12°C from 0–50°C) was inserted 10 cm (4.5 inches) into the rectum and taped to the tail for the duration of the anesthetic event. All temperatures were measured by the same evaluator.

### Anesthetic protocol

Clinically used premedications were selected due to their differential effects on vascular tone. Each dog was sedated with either 1) acepromazine (0.05 mg/kg) intramuscularly to produce vasodilation via alpha-1 adrenergic receptor blockade or 2) dexmedetomidine (5 mcg/kg) intramuscularly to produce vasoconstriction via alpha-2 adrenergic receptor activation, followed in 15 minutes by placement of a 22-gauge intravenous (IV) catheter in the right cephalic vein (Zoetis, Inc., Kalamazoo, MI) [16,17]. Doses were selected based on clinical recommendations used at the primary institution. Propofol was administered IV at a rate of approximately 1 mL/20 seconds until loss of the palpebral reflex and sufficient jaw relaxation was noted to facilitate orotracheal intubation. General anesthesia was maintained using isoflurane delivered in 100% oxygen (~1 L/min) targeting an end tidal isoflurane (ETiso) of 1.3%. Mechanical ventilation was performed with a tidal volume of approximately 10 mL/kg, respiratory rate of 8–12 beats per minute (bpm), and peak inspiratory pressure of 8–10 cm H_2_O to maintain end tidal carbon dioxide (ETCO_2_) between 35–45 mmHg. Dogs were positioned in dorsal recumbency on a thin cotton pad covering a circulating water blanket. A #3 sized blood pressure cuff measuring approximately 40% of the limb circumference was placed proximal to the tarsus for systolic (SAP), diastolic (DAP), and mean blood pressure (MAP) measurement to indirectly estimate the degree of vasodilation or vasoconstriction as part of the complimentary study [15]. Dogs were monitored with a pulse oximeter (SpO_2_), electrocardiogram (ECG), capnometer (ETCO_2_), and rectal thermometer connected to a multiparameter monitor (Datascope, Passport 2, Mindray North America, Mahwah, NJ). Pulse rate, respiratory rate, SpO_2_, ETCO_2_, SAP, MAP, DAP, ETiso, and oxygen flow rate were recorded every five minutes, while rectal and CORE sensor temperatures were recorded every three minutes. Room temperature was maintained at 21.1–21.7˚C (70–71˚F) and unwarmed IV balanced crystalloids (Plasma-Lyte A, Baxter, Round Lake, IL; 5 mL/kg/hr) were administered.

Temperature readings were recorded at baseline and throughout the experimental protocol. Each dog included in this study had active warming with a circulating water blanket as well as a forced-air warming blanket throughout the procedure, noted in the complimentary study as “conventional” heating devices. Dogs were maintained under general anesthesia for approximately two hours or until dogs became hypothermic (<36°C) or hyperthermic (>39.5°C); if they met these criteria, they were maintained under general anesthesia for an additional ten minutes prior to discontinuing inhalant administration. At that point in time, dogs recovered from anesthesia, and temperature monitoring was discontinued.

### Statistical analyses

In consultation with a statistician, statistical analyses were performed using SAS software (version 9.4; SAS Institute Inc., Cary, NC, USA). Descriptive statistics were summarized and reported. Relationships between rectal temperature and the CORE temperature sensor placed on clipped and unclipped skin were evaluated using linear mixed-effect regression models to account for repeated measures, and repeated-measures correlation coefficients (*r*_*rm*_) were calculated. On visual inspection, all data revealed a bell-shaped curve and the residual plots from the linear mixed-effect model did not show violations of normal assumption. Regression lines (solid lines) and corresponding 95% prediction bands were reported.

Agreement between rectal thermometry and each alternative method was assessed using Bland–Altman (BA) analysis for repeated measures [18]. The mean bias was defined as the mean difference between paired measurements and is illustrated by the short, dashed line parallel to the x-axis relative to the line of zero difference (solid line). The 95% limits of agreement (LOA), calculated as the mean difference ± 1.96 × standard deviation (SD) of the differences, are shown as long dashed lines. Under the assumption of normally distributed differences, 95% of paired differences are expected to lie within these limits. They are used to assess how well the two thermometric methods agree using a maximum clinically acceptable difference in temperature pre-determined at 0.3°C, similar to previously published studies [19,20].

Inter-rater reliability was assessed using the Shrout-Fleiss intraclass correlation coefficients (ICC; Tables 1 and 2) and 95% confidence intervals (CI; Table 2). ICC values were interpreted as follows: > 0.90, excellent reliability; 0.75–0.90, good reliability; 0.50–0.74, moderate reliability; and < 0.50, poor reliability [21,22]. To translate statistical reliability into practical clinical utility, the calculated ICC values were mapped to qualitative clinical applicability descriptions as follows: ICC values less than 0.50 were deemed unacceptable for clinical use due to inconsistency; values from 0.50 to 0.75 were restricted to preliminary screening purposes only; values from 0.75 to 0.90 were classified as dependable for routine clinical decisions; and values exceeding 0.90 were designated as meeting the standards required for independent, diagnostic-grade assessments.

**Table 1:** Shrout-Fleiss reliability intraclass correlation coefficients [21,22].

ICC Reliability Value	Clinical Applicability
< 0.5: Poor	*Unacceptable for clinical use.* Inconsistent measurements make the method studied unreliable for guiding clinical decisions.
0.5-0.75: Moderate	*Use only with extreme caution.* Limited consistency between the studied and comparative methods restricts its use to clinical screening and not decision-making.
0.75-0.9: Good	*Suitable for clinical practice.* Strong consistency between the studied and comparative methods makes it highly dependable for making clinical decisions.
> 0.9: Excellent	*Diagnostic-grade reliability.* The method studied meets the stringent standards required for critical clinical diagnostics and independent assessments.

**Table 2:** Thermometric method descriptive statistics and correlations.

Method	Sample Size	Mean ± Standard Deviation (°C)	Median [Minimum, Maximum] (°C)	Shrout-Fleiss ICC [95% CI]: Rectal-Alternative Method	Correlation Coefficient for Repeated Measures (*r*_*rm*_) [95%CI]
**Acepromazine**					
Rectal	125	37.7 ± 0.6	37.5 [36.9, 39.4]		
Clipped CORE	125	38.4 ±0.5	38.3 [37.7, 39.4]	0.78 [0.70, 0.84] Good	0.81 [0.58, 1.00]; P < 0.0001
Unclipped CORE	125	37.8 ± 0.6	37.6 [37.1, 39.3]	0.85 [0.80, 0.89] Good	0.79 [0.54, 1.00]; P < 0.0001
**Dexmedetomidine**					
Rectal	127	38.0 ± 0.7	38.1 [36.8, 39.3]		
Clipped CORE	125	38.5 ± 0.7	38.5 [36.8, 40.3]	0.28 [0.11, 0.43] Poor	0.30 [0.17, 0.43]; P < 0.0001
Unclipped CORE	125	38.2 ± 0.7	38.1 [36.5, 39.9]	0.88 [0.83, 0.91] Good	0.48 [0.38, 0.58]; P < 0.0001

The study was powered to detect clinically meaningful agreement between temperature measurement methods since only 3 dogs were used. Linear mixed-effect models were used to account for within-subject correlation and non-independence, by including the dog's identification number as a random effect, acknowledging that measurements from the same dog are grouped together. A concordance (new measurement method versus gold standard method) design was used to test whether the concordance correlation coefficient (CCC) is greater than 0.50 (H0: CCC ≤ 0.50 versus H1: CCC > 0.50) as a value of < 0.50 indicates poor agreement between methods. Comparisons were made using the appropriate Lin's Concordance Correlation Coefficient test, with a Type I error rate (α) of 0.05. To detect a concordance correlation coefficient of 0.7 with a sample size of 135 as used in the companion study [15], the power is 0.98. The power was computed using Power Analysis and Sample Size (PASS) software (2025, version 25.0.2).

## Results

Temperatures were recorded with all three methods (rectal, clipped, and unclipped CORE) every three minutes for up to two hours in dogs or until clinically meaningful hypothermia or hyperthermia was detected, resulting in 125–127 repeated measures samples per drug (acepromazine or dexmedetomidine; Table 2).

From the linear mixed-effect model, temperatures (least squared means ± standard error) in anesthetized dogs following dexmedetomidine were higher than following acepromazine in the rectal (38.1 ± 0.3°C versus 37.7 ± 0.3°C; P < 0.0001) and unclipped CORE (38.2 ± 0.3°C versus 37.8 ± 0.3°C; P < 0.0001) groups. There were no statistical differences between treatments in the clipped CORE group (38.5 ± 0.3°C versus 38.4 ± 0.3°C; P = 0.35).

Based on the correlation coefficient for repeated measures (*r*_*rm*_) and the Shrout-Fleiss intraclass correlation (ICC; Table 2), following acepromazine and inhalant anesthetic administration in dogs, there was good reliability and significant correlation between rectal and clipped (Fig 1) or unclipped (Fig 2) CORE temperatures, indicating that the CORE measurements were somewhat consistent with digital rectal thermometry. When dexmedetomidine was used prior to inhalant anesthesia in dogs, the clipped CORE temperatures (Fig 3) showed poor reliability to predict rectal temperatures, although they were still linearly correlated. The unclipped CORE temperatures (Fig 4) had good reliability to predict rectal temperatures and significant linear correlation. No CORE measurements showed excellent reliability (ICC > 0.9) with rectal temperatures in this population of anesthetized dogs although the unclipped CORE values nearly approached an ICC = 0.9.

**Fig 1:**
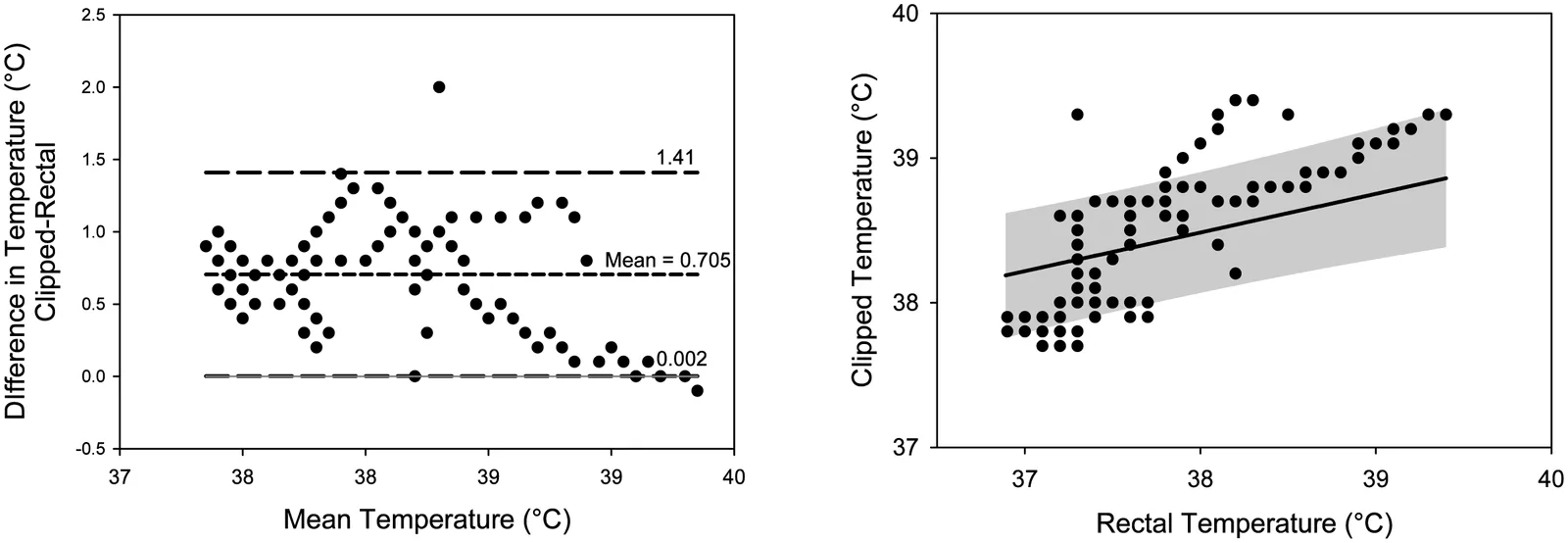
Regression and Bland-Altman plots assessing correlation and agreement of rectal and clipped CORE temperatures following acepromazine premedication and inhalant anesthesia. Left: The fitted regression line (black line) and 95% prediction band (gray curves) depict a good reliability and statistically significant correlation between rectal and clipped CORE thermometric methods. Right: The Bland-Altman plot shows the mean difference (short, dashed line) and 95% upper and lower limits of agreement (long dashed line) depicting a weak agreement (< 0.3°C) between rectal and clipped CORE temperatures.

**Fig 2:**
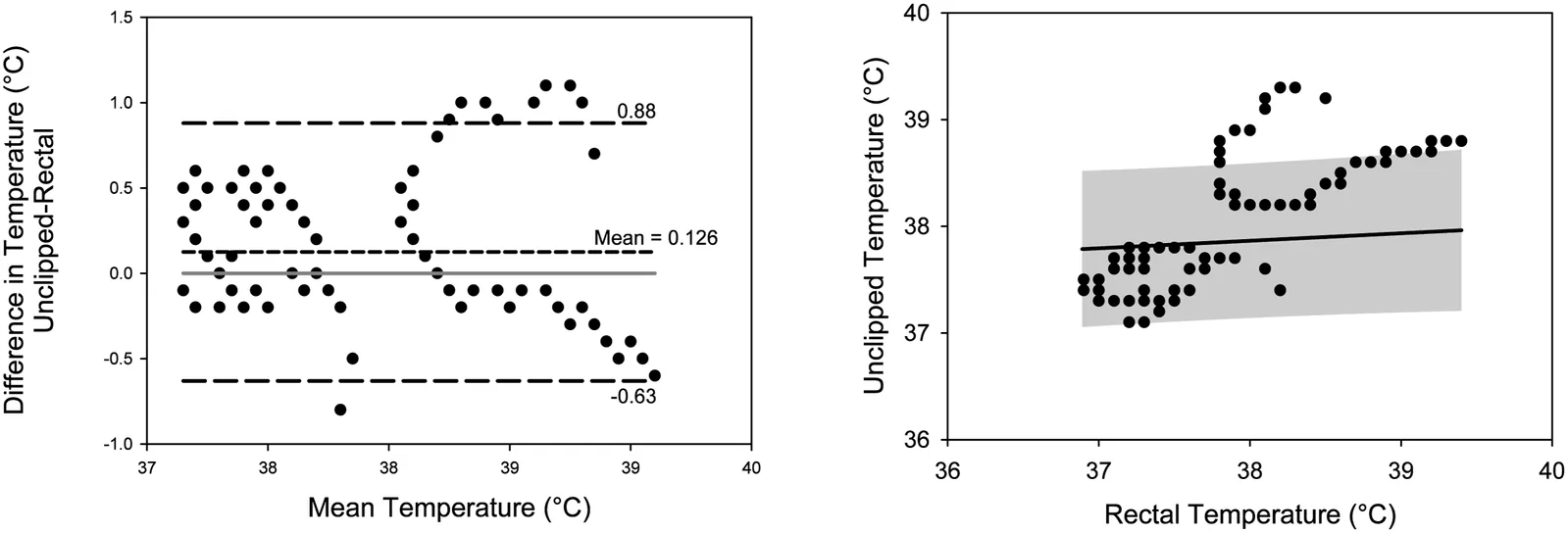
Regression and Bland-Altman plots assessing correlation and agreement of rectal and unlipped CORE temperatures following acepromazine premedication and inhalant anesthesia. Left: The fitted regression line (black line) and 95% prediction band (gray curves) depict a good reliability and statistically significant correlation between rectal and unclipped CORE thermometric methods. Right: The Bland-Altman plot shows the mean difference (short, dashed line) and 95% upper and lower limits of agreement (long dashed line) depicting a clinically acceptable agreement (< 0.3°C) between rectal and unclipped CORE temperatures.

**Fig 3:**
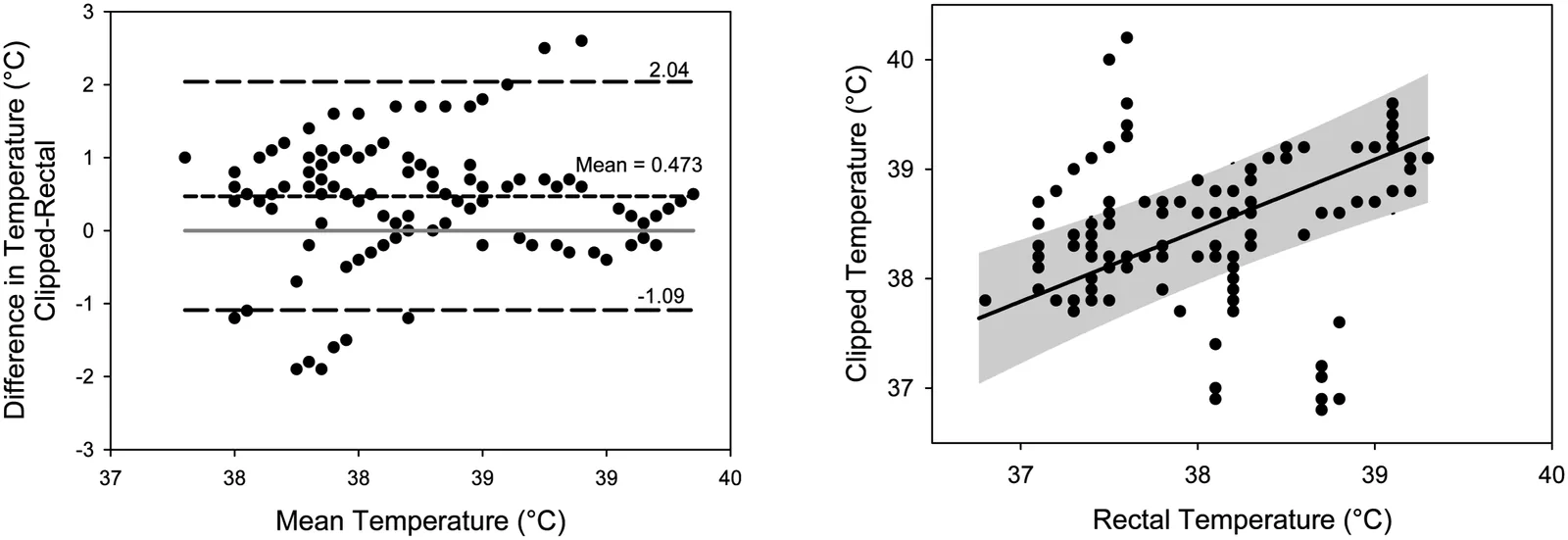
Regression and Bland-Altman plots assessing correlation and agreement of rectal and clipped CORE temperatures following dexmedetomidine premedication and inhalant anesthesia. Left: The fitted regression line (black line) and 95% prediction band (gray curves) depict a poor reliability, yet statistically significant correlation between rectal and clipped CORE thermometric methods. Right: The Bland-Altman plot shows the mean difference (short, dashed line) and 95% upper and lower limits of agreement (long dashed line) depicting a weak agreement (> 0.3°C) between rectal and clipped CORE temperatures.

**Fig 4:**
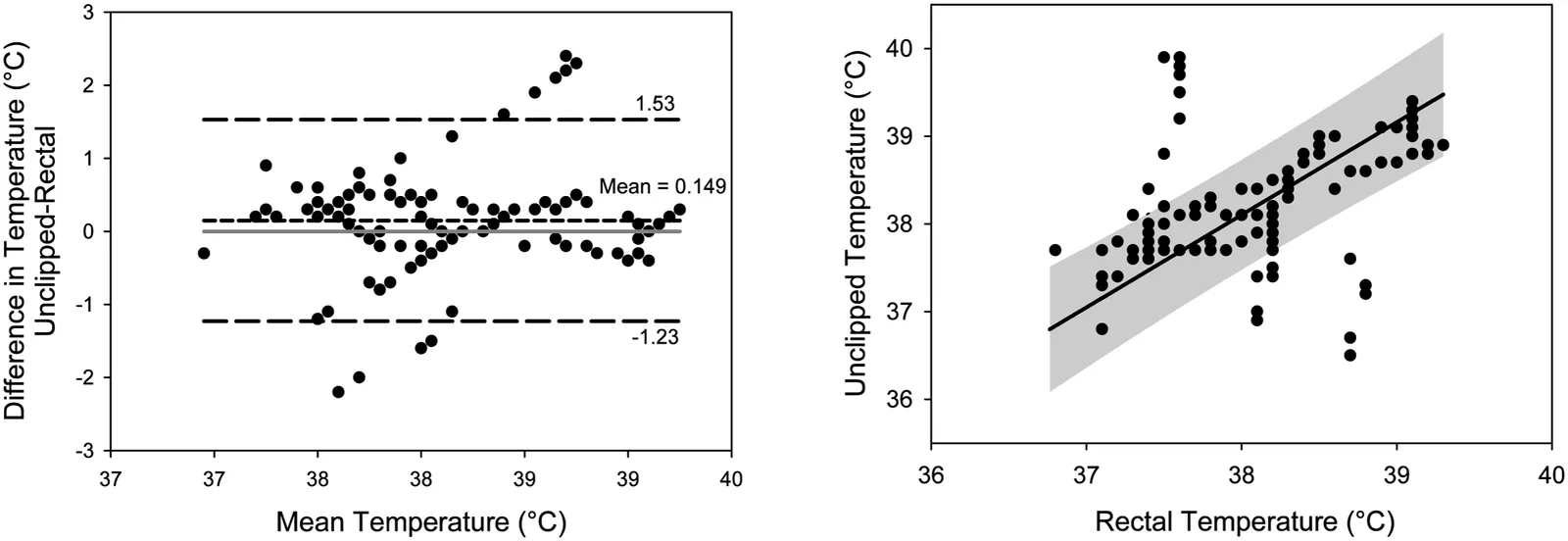
Regression and Bland-Altman plots assessing correlation and agreement of rectal and unclipped CORE temperatures following dexmedetomidine premedication and inhalant anesthesia. Left: The fitted regression line (black line) and 95% prediction band (gray curves) depict a good reliability and statistically significant correlation between rectal and unclipped CORE thermometric methods. Right: The Bland-Altman plot shows the mean difference (short, dashed line) and 95% upper and lower limits of agreement (long dashed line) depicting a clinically acceptable agreement (< 0.3°C) between rectal and unclipped CORE temperatures.

The maximum clinically acceptable difference between thermometric methods was pre-determined to be 0.3°C and only the mean difference between rectal and unclipped CORE temperatures in the acepromazine (mean difference [95% CI] = 0.126 [0.058, 0.194] °C; Fig 2) and dexmedetomidine groups (mean difference [95% CI] = 0.149 [0.025, 0.273] °C; Fig 4) showed strong agreement. Agreement between rectal and clipped CORE measurements following acepromazine (mean difference [95% CI] = 0.705 [0.642, 0.768] °C and dexmedetomidine (mean difference [95% CI] = 0.473 [0.332, 0.614] ° C did not meet this criterion (Figs 1 and 3).

## Discussion

This preliminary study evaluated the ability of the CORE sensor to estimate body temperature in anesthetized dogs premedicated with dexmedetomidine or acepromazine. Across the repeated measurements in each group, dogs who received dexmedetomidine had consistently higher body temperatures compared to those who received acepromazine when measured rectally or with the unclipped CORE sensor. Significant linear correlations were identified between rectal and either clipped or unclipped CORE methods in both treatment groups, indicating that CORE measurements generally followed rectal temperature trends over time. However, although unclipped CORE temperatures following acepromazine and dexmedetomidine and clipped CORE temperatures following acepromazine showed good reliability with rectal temperatures in anesthetized dogs, clinical agreement was only acceptable (< 0.3°C) between rectal and unclipped CORE temperatures; clipped CORE temperatures did not meet this criterion during anesthesia and concurrent warming techniques. Overall, when the CORE sensor is applied to unclipped skin, temperature data obtained may be suitable as an alternative, non-invasive thermometric technique in anesthetized dogs. However, given the limited number of dogs, these results should be treated as exploratory rather than definitive, serving as a basis for future, larger-scale validation studies.

Although all CORE measurements, whether acepromazine or dexmedetomidine was used as a premedicant, had statistically significant repeated measures correlations, this value simply implies the strength of a linear relationship (i.e., when one value changes, the other changes proportionally). Because correlation only measures association and ignores systematic bias, meaning two methods can be perfectly correlated even if one consistently reads much higher or lower than the other, it provides little information on actual agreement between measurements. The Shrout-Fleiss ICC, by contrast, measures the exact absolute agreement between methods by accounting for both correlation and systematic shifts. For example, it is a ratio that compares the true biological variation to the total variation, which includes measurement error and method-related bias. Thus, following acepromazine, the clipped and unclipped CORE method, as well as the unclipped method in the dexmedetomidine group have good reliability. This indicates that the method reads consistently and discriminatively, and if a clinical difference in temperatures is present, it is due to a true difference and not due to errors in the measurement process. These statistical inferences, which produce unitless measures of relative agreement, differ from the Bland-Altman analysis used to assess acceptable clinical differences. While the ICC evaluates reliability relative to overall population variability, the Bland-Altman method quantifies the actual systematic bias (mean difference) and limits of agreement in the original clinical units (°C), directly helping clinicians determine appropriate treatments for patient care. For example, an acceptable clinical difference of 0.3°C was selected based on previously published studies, veterinary expert (Diplomate) consensus, the tight range of normal canine body temperatures, and clinical consequences of misdiagnosing thermoregulatory disturbances since therapeutic interventions may be used in patients (intentional cooling or warming) if temperatures deviate from this value [15,19,20,23].

Our results only partially supported our hypotheses. Although the CORE sensor placed on clipped and unclipped skin correlated with rectal temperatures and demonstrated reliability in some anesthetized conditions in dogs’ measurements, the sensor was most reliable and had better clinical validity when used on unclipped skin, despite premedication. Although it was hypothesized that a thermometric sensor placed directly on clipped skin would have better contact and improve agreement with rectal temperatures, this was not the case. The CORE sensor placement on the neck separated two thermal environments – the ambient air and the skin underneath. To accurately read the body temperature, the contact resistance between the skin and sensor must be low while the environmental resistance between the air and sensor must be high to ensure that body versus air temperature is measured. Since intact fur is a natural insulator, it may help to increase environmental resistance, preventing the circulating air from reaching the sensor and consequently giving erroneous body temperature readings. We speculate that clipping the fur may have made the environmental resistance so low that the sensor was exposed to the warm air from the forced air warmer, creating inaccurately increased body temperature readings. Thus, clipping may have changed both contact and insulation quality. Although the clipped side allowed closer sensor-to-skin coupling, the unclipped side likely retained some body heat and created an insulating air gap as well, resulting in more valid readings. We also did not directly measure differences in microenvironmental temperature, local airflow, or contact pressure which could also explain side-to-side differences; these require further investigation.

Dexmedetomidine-induced vasoconstriction may also have influenced results. For example, non-invasive blood pressures recorded from these same dogs suggested a clinically detectable degree of vasodilation (acepromazine) and vasoconstriction (dexmedetomidine) [15]. Although premedication and had minimal effect on the clinical validity of the CORE sensor, the reliability, as measured by the ICC, was poor in the clipped CORE group compared with rectal temperatures following dexmedetomidine, suggesting that vasoconstriction and increased exposure to the environment may have altered the microclimate to a greater extent than in unclipped dogs and dogs administered acepromazine.

Several methods of core body temperature monitoring under general anesthesia have been evaluated in humans in addition to animals. The gold standard for monitoring core temperature, defined as the temperature of internal organs in humans, is obtained through placement of a pulmonary artery catheter, typically a Swan-Ganz [24]. While this method can be employed in veterinary species, it is technically difficult and requires special equipment not often used in clinical veterinary medicine. In dogs, rectal and esophageal thermometry are considered the most reliable methods for estimating core temperature under general anesthesia and are used interchangeably; however, these methods are not considered gold standard for measuring core body temperature [25]. The current study demonstrated that measuring temperature via the CORE sensor placed on unclipped skin superficial to the jugular vein is likely a clinically valid method to estimate core temperature trends in healthy dogs under general anesthesia when compared with rectal thermometry; use on clipped areas cannot be recommended.

A key limitation of this study is the small sample size (n = 3 dogs). While we analyzed more than 120 temperature measurements, the reliance on a small cohort introduces the issue of pseudoreplication, as repeated measures do not substitute for independent biological replicates. Although these repeated assessments increase our power to observe temporal trends, they limit our ability to generalize the findings. As such, these results are best viewed as a pilot validation, serving as a foundation for future, larger-scale trials in anesthetized canine patients.

Other study limitations consider both device-specific constraints and the physiological effects of the anesthetic protocols utilized for this study. For example, the CORE sensor relies on heat flux and skin temperature to estimate core body temperature. Unlike humans, dogs have fur for insulation and have possible breed differences that may influence heat transfer, potentially adding to measurement variability [26]. In addition, the CORE sensor has not yet been calibrated for veterinary species; however, data presented here may help to validate new algorithms for monitoring core temperature using the sensor.

The pharmacodynamics associated with dexmedetomidine or acepromazine may also affect thermoregulation [6,27], and can widen the potential gradient between core and peripheral temperatures. The CORE sensor accuracy zone depends on peripheral heat exchange; therefore, drug-induced changes to the periphery may affect its accuracy. The CORE sensor accuracy range reported by the manufacturer is ± 0.26°C from −20–45°C which is wider than the digital rectal thermometer accuracy range of ±0.12°C from 0 to 50°C [12,28].

Furthermore, the CORE sensor's reliance on skin contact and susceptibility to ambient temperature introduce potential measurement variables. For example, active patient warming may have created a local microclimate artifact that influenced readings on the clipped side. This issue was likely compounded by a lack of bilateral randomization in sensor placement. While alternative anatomical sites might yield different levels of agreement with rectal temperature, we did not employ the chest/torso placement recommended in humans [29]. However, we minimized confounding variation by standardizing the sensor's location across all dogs and timepoints

The CORE temperature measurements may also differ in dogs not undergoing general anesthesia with inhalant anesthetics. General anesthesia causes hypothermia by impairing hypothalamic thermoregulation and redistribution of warm blood from the core to the periphery; hypothermia occurs quickly after induction of general anesthesia [15,30]. Conscious or minimally sedated dogs may have better homeostatic control of thermoregulation and results may differ.

This pilot investigation consisted of a very small sample size which increases risk of both Type I and Type II errors. Although it was performed in conjunction with a statistician in a cross over design to improve statistical power, larger investigations further defining the utility of the CORE sensor to reliably detect clinical changes in body temperature in dogs are required.

## Conclusions

This pilot study demonstrated that monitoring temperature under general anesthesia with the CORE sensor provided reliable data when measuring core temperature trends in rectal thermometry, especially when placed on unclipped skin although the techniques cannot be considered interchangeable at this time. When further studies utilizing larger, clinically diverse populations, conscious dogs, other pharmacologic agents, and other sensor locations are performed, broader clinical recommendations for temperature regulation may be more reliably based when derived from the CORE sensor on unclipped areas of the neck in anesthetized dogs following acepromazine or dexmedetomidine premedication. However, until further investigations are completed, clinical decision making should be made cautiously if solely using the CORE sensor.

## Supporting information

S1 Dataset Original data used for manuscript preparation and statistical inferences. (XLSX)
